# Bronchial and Systemic Relationships of *Haemophilus* in Chronic Obstructive Pulmonary Disease

**DOI:** 10.3390/ijms27083416

**Published:** 2026-04-10

**Authors:** Eduard Monsó, Carme Casadevall, Sara Quero, Sergi Pascual-Guàrdia, César Jésse Enríquez-Rodríguez, Laura Millares, Concepción Montón, Rosa Faner, Silvia Capilla, Luis Miguel Seijo, Ady Castro-Acosta, Carlos Alvarez-Martínez, Oriol Sibila, Germán Peces-Barba, Borja G. Cosio, Alvar Agustí, Joaquim Gea

**Affiliations:** 1 Institut d’ Investigació i Innovació Parc Taulí (I3PT-CERCA), 08208 Sabadell, Spain; sara.queroe.blanca@gmail.com (S.Q.); lmillares@iconcologia.net (L.M.); 2 Barcelona Respiratory Network, 08009 Barcelona, Spain; carme.casadevall@upf.edu (C.C.); spascual@parcdesalutmar.cat (S.P.-G.); cesarjesse.enriquez01@alumni.upf.edu (C.J.E.-R.); rfaner@recerca.clinic.cat (R.F.); osibila@clinic.cat (O.S.); aagusti@clinic.cat (A.A.); jgea@hmar.cat (J.G.); 3 Department of Respiratory Medicine, Hospital Del Mar, Hospital Del Mar Research Institute, MELIS, Universitat Pompeu Fabra (UPF), 08003 Barcelona, Spain; 4 Centro de Investigación Biomédica en Red, Área de Enfermedades Respiratorias (CIBERES), Instituto de Salud Carlos III, 28029 Madrid, Spain; luis.seijo@fjd.es (L.M.S.); ady@h12o.es (A.C.-A.); carlosjose.alvarez@salud.madrid.org (C.A.-M.); gpeces@fjd.es (G.P.-B.);; 5 Department of Respiratory Medicine, Hospital Universitari Parc Taulí, Universitat Autònoma de Barcelona, 08208 Sabadell, Spain; cmonton@tauli.cat; 6 Health Services Research on Chronic Diseases Network-REDISSEC, 48960 Galdakao, Spain; 7 Departament Biomedicina, Fundació Clínic per la Recerca Biomèdica, Universitat de Barcelona, 08036 Barcelona, Spain; 8 Department of Microbiology, Hospital Universitari Parc Taulí, Universitat Autònoma de Barcelona, 08208 Sabadell, Spain; scapilla@tauli.cat; 9 Department of Respiratory Medicine, Clínica Universidad de Navarra, 28027 Madrid, Spain; 10 Department of Respiratory Medicine, Hospital 12 De Octubre, 28041 Madrid, Spain; 11 Department of Respiratory Medicine, Institut Clínic de Respiratori, Hospital Clínic, Fundació Clínic per la Recerca Biomèdica, Universitat de Barcelona, 08036 Barcelona, Spain; 12 Department of Respiratory Medicine, Fundación Jiménez Díaz, Clínica Universidad de Navarra, Universidad Autónoma de Madrid, 28040 Madrid, Spain; 13 Department of Respiratory Medicine, Hospital Son Espases, Instituto de Investigación Sanitaria de Palma (IdISBa), Universitat de Les Illes Balears, 07120 Palma de Mallorca, Spain

**Keywords:** COPD, exacerbation, admission, microbiome, *Haemophilus parainfluenzae*, *Haemophilus influenzae*, interleukin-8

## Abstract

The aim of the study was to assess the microbial composition of bronchial secretions in chronic obstructive pulmonary disease (COPD), focusing on the impact of the exacerbation patterns on the common components of the respiratory flora and their relationship with inflammatory proteins. A total of 72 clinically stable COPD patients provided sputum and blood samples for 16S rRNA gene amplification and peripheral biomarkers. Beta-diversity analyses of the bronchial microbiome showed significant differences between infrequent and frequent (≥2) exacerbators (*p* = 0.001). *Haemophilus* was underrepresented in frequent exacerbators (relative abundance [RA] 0.07 [0.003–0.31] vs. 0.24 [0.06–2.36], *p* = 0.02) while the presence of *Pseudomonas* was increased (7.70 [0.66–11.68] vs. 1.11 [0.37–2.88], *p* = 0.01). Eight common taxa, *Prevotella*, *Moryella*, *Atopobium*, *Megasphaera*, *Parvimonas*, *Veillonella*, *Bulleidia* and *Selenomonas*, showed significant decreases in their RAs when exacerbations required hospitalization. RAs of *Haemophilus* and eight common taxa were positively correlated (*p* < 0.01). Among them, *Porphyromonas*, *Leptotrichia* and *Selenomonas* showed a negative correlation with blood interleukin-8 (IL-8) (*p* < 0.01) and an equivalent correlation was found for *Haemophilus parainfluenzae*. Frequent exacerbations cause a decrease in the RA of *Haemophilus* and have a more extensive impact when hospitalization is required. The RAs of common bronchial bacteria were closely related and some of them were inversely associated with blood IL-8 levels.

## 1. Introduction

*Haemophilus*, *Prevotella*, *Rothia*, *Streptococcus* and *Veillonella* are main components of the respiratory microbiome of chronic obstructive pulmonary disease (COPD) [1,2,3,4,5,6], but significant changes in this microbial flora have been reported in severe patients, who often report frequent exacerbations that require chronic treatment with inhaled corticosteroids [7]. A decline in most of these common components of the respiratory microbiome has been described in this clinical situation [2,4,8], which some authors have associated with an overrepresentation of *Haemophilus influenzae* in the bronchial microbiome [6,9,10,11]. *Haemophilus* taxon relative abundances (RAs) below 5% are common in stable COPD patients [2,3,4,5], but some studies have observed significant increases in this taxon in population samples enriched with frequent exacerbators, where *Haemophilus* attained RAs over 20%, mostly attributable to *Haemophilus influenzae* [6]. These studies had included a high proportion of patients with three or more exacerbations in the previous year, who may not be representative of the general COPD population.

*Haemophilus parainfluenzae* is the main *Haemophilus* taxon in the respiratory microbiome in healthy people and stable COPD patients with moderate disease [12,13,14], and this microorganism has not been associated with a significant bronchial inflammatory response [14,15]. Choi and cols. [16] had analyzed the presence of *H. parainfluenzae* in the bronchial microbiome of COPD patients, both in clinical stability and during exacerbations, and reported that stable patients showed significantly higher RAs of *H. parainfluenzae* and *Neisseria perflava*, and equivalent results have been reported by other authors [17]. The appearance of frequent exacerbations in the natural history of COPD favors a shift between *H. parainfluenzae* and *H. influenzae*, with an increase in the abundance of this second microorganism in patients who exacerbate frequently [17]. This shift may be favored by the recurrent antibiotic treatments required by COPD patients with frequent exacerbations, which have a direct impact on *H. parainfluenzae*, decreasing its abundance in the respiratory tract [13]. Any partial substitution of *H. parainfluenzae* by *H. influenzae* would have an impact in bronchial inflammation patterns, considering that *H. influenzae* is able to induce local cellular inflammation [17], and the release of cytokines such as interleukin-8 (IL-8) in the bronchial tree [11,18,19]. The appearance of *H. influenzae* in bronchial secretions of COPD patients also determines different changes in the bronchial mucosa that favor the bronchial adherence of the microorganism to the epithelial cells, with biofilm formation [20,21]. *H. influenzae* is able to express biofilm-formation genes promoting the appearance of epithelial and mucus-binding adhesins [22]. *H. influenzae*-induced biofilms exhibit extreme tolerance to antimicrobial treatments and favor the chronic colonization of the bronchial tree by the microorganism and other potentially pathogenic bacteria [23], which increase their RAs in the bronchial microbiome. These observations suggest that the effects of *H. parainfluenzae* and *H. influenzae* on the inflammatory pathways active in COPD may be different. Accordingly, to investigate the weight of the different *Haemophilus* species in bronchial secretions of COPD patients, determining the impact of exacerbation patterns on this composition is a relevant topic, considering that the potential impacts of the different *Haemophilus* species on the local inflammatory response may be different.

The aim of the present study was to examine the effects of moderate and severe exacerbations on the bronchial microbiome of COPD patients, focusing on the common components of the respiratory flora, and, among them, the *Haemophilus* genus, assessing microbial interactions and their relationships with IL-8 at a systemic level.

## 2. Results

### 2.1. Clinical Characteristics, Bronchial Microbiome and Protein Biomarkers

Participants had a mean age of 68 (SD 7.9) years and a median forced expiratory volume in the first second, expressed as percentage from the reference value (FEV1%), of 44 (IQR 33–60). The enrolled patients reported a median of 1 (IQR 0–2) exacerbations and 0 (IQR 0–1) hospitalizations due to their respiratory disease during the previous year (Table 1), and these two clinical variables were significantly correlated (Spearman rho coefficient: 0.81, *p* < 0.001). All patients reporting ≥2 acute episodes the previous year were considered frequent exacerbators, and required at least one hospitalization (20/20, 100%), but 11/52 (21.2%) participants who suffered only one exacerbation also required an admission for their acute episode, a finding that justifies the independent management of these two variables. Previous exacerbation and hospitalization frequencies were inversely correlated with FEV_1_% (Spearman rho coefficient: −0.45, *p* < 0.001; and −0.34, *p* = 0.003).

Six phyla and 20 genera from the 13 phyla and 156 genera identified in sputum samples showed RAs ≥ 0.1% and appeared in at least 80% of the enrolled patients. These taxa were considered the common bronchial flora of COPD for the purposes of the study and were included in the subsequent analyses (Table 2 and Supplementary Table S1). Forty-seven out of 58 analyzed proteins attained blood values over their limit of detection (Supplementary Table S2).

### 2.2. Relationships Between Bronchial Microbiome and Clinical Patterns

**Figure 1:**
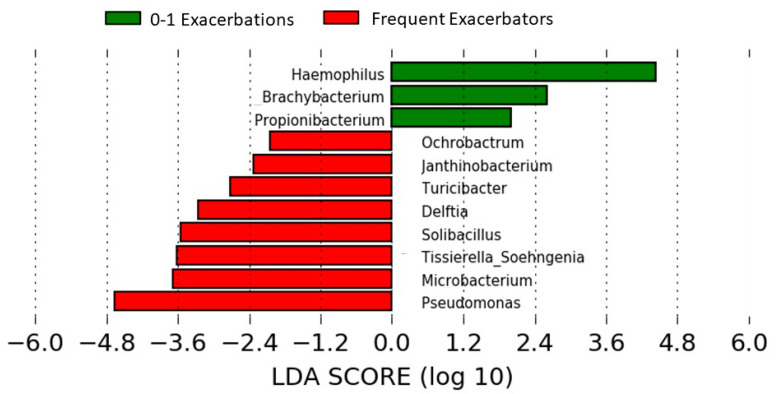
Effect of frequent exacerbations on microbiome diversity. Comparison of patients reporting two or more exacerbations the previous year with infrequent exacerbators. LEfSe analysis.

Beta-diversity analyses considering the bronchial flora as a whole showed a significant difference between frequent and infrequent exacerbators (*p* = 0.001, PERMANOVA on unweighted UniFrac). *Pseudomonas* showed higher RA in frequent exacerbators (median [IQR], 7.70 [0.66–11.68] vs. 1.11 [0.37–2.88], *p* = 0.01, Mann–Whitney U test), while *Haemophilus* was underrepresented in this subgroup (0.07 [0.00–0.31] vs. 0.24 [0.06–2.36], *p* = 0.02, Mann–Whitney U test). In LEfSe analyses these differences attained a significant effect size (Figure 1).

**Figure 2:**
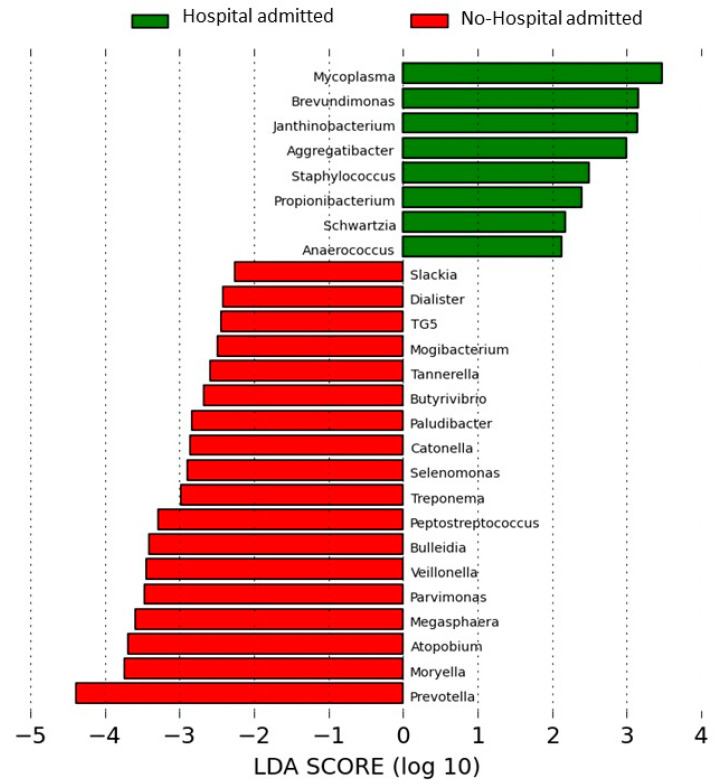
Effect of previous hospitalization on microbiome diversity. Comparison of hospital-admitted and non-admitted patients in the previous year. LEfSe analysis.

A significant difference in beta-diversities was also observed when admitted and non-admitted patients were compared (*p* = 0.004, PERMANOVA on unweighted unifrac), with a wide range of common taxa showing significant reductions in their RAs in sputum samples from COPD patients who had required an admission the previous year (Table 3), with a high effect size in LEfSe analysis (Figure 2). These findings confirm the wide effect of admissions on the bronchial flora.

### 2.3. Interbacterial Relationships in the Bronchial Microbiome

**Figure 3:**
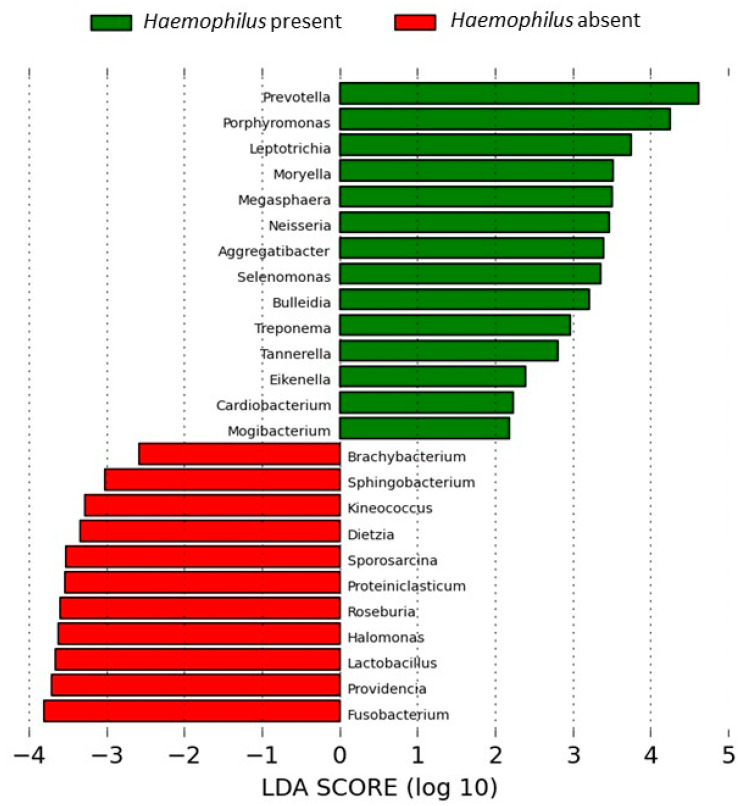
Taxa associated with *Haemophilus* genus. LEfSe analysis.

The presence of *Haemophilus* was significantly associated with higher RAs of *Prevotella* and six additional taxa from the common bronchial flora (*Porphyromonas*, *Leptotrichia*, *Moryella*, *Megasphaera*, *Selenomonas* and *Bulleidia*) (*p* < 0.05, Mann–Whitney U test). These overrepresentations showed a significant effect size in LEfSe analysis (Figure 3).

**Figure 4:**
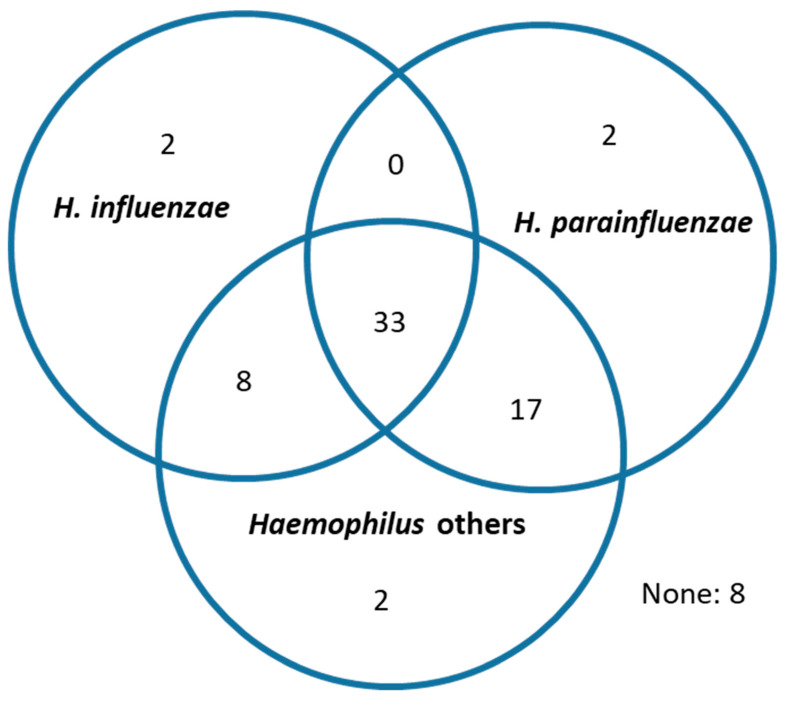
*Haemophilus* taxa present in bronchial secretions. “*Haemophilus* others” refers to species other than *influenzae* and *parainfluenzae* and taxa unidentified at species level (n = 72).

An identification of *Haemophilus* taxa at species level was partially attained for most patients. *Haemophilus influenzae* was identified in 43 (59.7%) while *H. parainfluenzae* and/or a mixture of other Haemophilus taxa was identified in 62 patients (86.1%). The genus was absent in eight of the enrolled patients (Table 4). The different *Haemophilus* taxa identified were simultaneously present in most patients (Figure 4), and the relationships observed between frequent exacerbations and *Haemophilus* cannot be specifically attributed to one particular species. The taxa associated with the *H. parainfluenzae* presence of *Haemophilus* showed a statistically significant relationship only with (*p* < 0.01, Mann–Whitney U test), and this relationship attained a significant effect size in LEfSe analysis.

### 2.4. Haemophilus-Related Taxa and Protein Biomarkers

*Haemophilus* RA was not significantly related to peripheral IL-8, but a negative and highly significant relationship was found between IL-8 level and the RAs of other components of the common flora such as *Porphyromonas*, *Leptotrichia* and *Selenomonas* (Spearman rho coefficient −0.3145 and −0.3255, *p* < 0.01, and −0.2658, *p* < 0.05, respectively). This association was also observed when the analysis of the different *Haemophilus* taxa was restricted to *H. parainfluenzae* (Spearman rho coefficient −0.3230, *p* < 0.01) (Supplementary Table S3).

## 3. Discussion

In the present COPD study, the clinical pattern of frequent exacerbations has been related to an increase in *Pseudomonas* RA in bronchial secretions, paralleled by a fall in the *Haemophilus* RA, while admission for one or more of these episodes was associated with a wider impact on the bronchial microbiome, with reductions in up to eight of the common taxa identified. Beta-diversity analyses confirmed the differences in the microbiomes in these clinical situations. *Porphyromonas*, *Leptotrichia* and *Selenomonas*, from the common genera in bronchial samples of the studied population, had RAs inversely related to IL-8 levels in peripheral blood, and this association was also found for *H. parainfluenzae*, a finding that may support an anti-inflammatory role for these components of the bronchial microbiome at a systemic level.

The common taxa present in the bronchial microbiome attained an RA approaching 50% in the studied population, with *Rothia*, *Prevotella*, *Granulicatella*, *Fusobacterium* and *Porphyromonas* emerging as the most representative genera, and a median RA of 0.19% for *Haemophilus*. This bronchial microbiome composition has a close similarity with results previously reported in moderate COPD patients [1,2,3,4,5]. Frequent exacerbators showed a significant impact on the bronchial microbiome, mainly attributable to an increase in the *Pseudomonas* genus and a decrease in the RA of *Haemophilus*, with significant effect sizes for both taxa in LEfSe analysis. An overrepresentation of *Pseudomonas* in bronchial secretions of patients reporting two or more exacerbations has been previously attributed to the severity of the disease and/or chronic colonization by this microorganism [25,26], but the relationships between *Haemophilus* and exacerbation patterns have given contradictory results in previous studies. Although RAs below 5% have been repeatedly reported in stable COPD patients [2,3,4,5], some studies have observed significant increases in this taxon in population samples enriched with frequent exacerbators, where *Haemophilus* had achieved RAs over 20% [6]. These studies included a high proportion of patients with three or more exacerbations in the previous year who may not be representative of the COPD population, and the characteristics of these population samples probably account for the observed differences.

In the present study a wide effect on the bronchial microbiome was observed in the participants who reported one or more hospital admissions in the previous year. As many as eight common taxa were significantly underrepresented in previously hospitalized patients, and this difference attained a significant effect size in LEfSe analysis. Frequent exacerbators and COPD patients with one or more admissions have been considered as a homogeneous population in previous publications and in some of the available guidelines [24,27], but the present results show that the effect of hospital admission on the bronchial flora is much wider than the changes in the microbiome induced by frequent exacerbations when an admission is not required. This finding suggests that considering frequent exacerbators and patients who require one or more admissions as a single group may be misleading.

*Haemophilus* was identified in nearly 90% of the participants in the present investigation, and a partial identification at species level was possible for most participants, who showed a predominance of *H. parainfluenzae* and other *Haemophilus* taxa over *H. influenzae*. Studies that have previously reported an overrepresentation of *Haemophilus* in the bronchial microbiome of COPD have focused on biased COPD populations with very high exacerbation frequencies, and have attributed the increase to *H. influenzae* [6,9,10]. The present study recruited COPD outpatients from different hospitals with a median exacerbation frequency of one episode in the previous year and included a quarter of frequent exacerbators, in closer similarity to the general COPD population [28]. The fall of *Haemophilus* taxa in bronchial secretions observed in patients reporting two or more exacerbations in the present analysis may suggest that species different from *H. influenzae* may first decrease when two or more exacerbations appear in the natural history of COPD patients. This pattern may move towards a progressive substitution by *H. influenzae* when the exacerbation frequency continues to increase at advanced stages, as suggested by Wang and cols., who have reported this partial substitution of *H. parainfluenzae* by *H. influenzae* when the severity of the COPD increases [17].

In the present study *Haemophilus* was associated with higher RAs of as many as seven common bronchial taxa, in agreement with previous analyses that have also shown a close association between *Haemophilus* and other genera commonly found in the respiratory system, such as *Leptotrichia* and *Porphyromonas* [27]. Various *Haemophilus* taxa were simultaneously present in most participants in the study, but *H. parainfluenzae* was the most prevalent and the only one showing a positive relationship with *Prevotella*, *Porphyromonas*, *Leptotrichia*, *Moryella*, *Megasphaera*, *Selenomonas* and *Bulleidia*, supporting the importance of *H. parainfluenzae* in the respiratory microbiome in stable COPD.

A negative and highly significant relationship was found between the RA of *Porphyromonas*, *Leptotrichia* and *Selenomonas* and the blood level of IL-8. The RA of *Haemophilus* in sputum was not significantly related to this protein biomarker, but *H. parainfluenzae* showed an equivalent correlation. Accordingly, the decline in these common respiratory taxa associated with frequent exacerbations may be associated with an increase in IL-8 in peripheral blood, which will enhance the systemic effects of this cytokine, which is a well-known chemoattractant for inflammatory cells [29]. The negative correlation between *H. parainfluenzae* and IL-8 levels is consistent with previous observations in bronchial secretions [15,17,30], which may be reversed in patients who change their bronchial microbiome to an overrepresentation of *H. influenzae*, who would increase their IL-8 levels in sputum [30,31]. Accordingly, any increase in the RA of *Haemophilus* in bronchial secretions may have opposite effects depending on the predominant species, with a potential protective effect when *H. parainfluenzae* is more prevalent, identifiable in peripheral samples, and an enhanced inflammatory response when *H. influenzae* attain an overrepresentation.

The performed analysis has some limitations that need to be pointed out. First, frequent exacerbation is a well-known clinical characteristic of severe COPD that requires chronic treatment with inhaled corticosteroids. Accordingly, the observed effect of frequent exacerbations on the bronchial microbiome may be attributable to the severity of the disease and/or its treatment. The marked collinearity between these variables makes it impossible to independently assess their effects on the bronchial flora. Second, the characterization of the *Haemophilus* taxon at species level using 16S rRNA gene sequencing, possible in most cases [32], sometimes could be difficult, and a partial misidentification at species level is a possibility that needs to be taken into account [33]. The taxonomy of the genus can be improved through quantitative PCR or culture, when enough sample is available [6,10]. The present study was based on 16S rRNA sequencing, without additional bronchial material available for extended species analyses. Although most of the *Haemophilus* taxa could be identified at species level, for a fraction of the *Haemophilus* operational taxonomic units the identification at that level was not attained, and these units were not considered for the analysis focused on the species level. Accordingly, the associations of *H. parainfluenzae* with the different variables analyzed in the present study must be considered exploratory.

## 4. Material and Methods

### 4.1. Study Design and Ethics

This cross-sectional analysis is part of the BIOMEPOC project, a prospective multicenter study described in detail elsewhere [34]. Briefly, the aim of BIOMEPOC is to identify the bronchial microbiome and circulating biomarkers in stable COPD patients to characterize more accurately the relationships between the respiratory flora and the different phenotypes of the disease. The study included a total of 269 patients and 83 controls. The protocol was approved by the local ethics committees from the seven teaching hospitals participating in the study. The investigation was conducted in accord with the Declaration of Helsinki and informed written consent was obtained from all participants.

### 4.2. Population

The present study was performed in a subsample of 72 consecutive patients enrolled at five of the participating hospitals (Hospital Parc Taulí, Hospital Clínic, Hospital 12 Octubre, Fundación Jimenez Díaz and Hospital Son Espases) from whom spontaneous sputum samples were available for microbiome analyses and blood samples for protein biomarker measurements obtained the same day. This study population comprised a cohort of stable COPD patients who were current or former smokers (≥10 pack-year) and were attending the outpatient clinics of the participating hospitals between 2014 and 2016. Diagnosis of COPD and its severity was established in accordance with the Global Initiative for Chronic Obstructive Lung Disease (GOLD) criteria [24]. Exclusion criteria were age ≤ 40 years, a lifetime diagnosis of asthma, cystic fibrosis, bronchiectasis or cancer, administration of long-term treatment with oral corticosteroids or immunosuppressants, any comorbidity limiting cognitive capabilities, one acute episode severe enough to require more than 30 days of hospitalization in the previous year and/or unstable disease which required short-term antibiotics and/or corticosteroids during the previous three months.

### 4.3. Variables and Measurements

Sociodemographic data were recorded by specific questionnaires. Lung function values during stability were obtained from the most recently available forced spirometry with reversibility testing performed in accordance with standard techniques. Episodes of increased dyspnea, sputum production and/or purulence during the previous year were identified and considered as exacerbations when treated with antibiotics and/or corticosteroids [35]. Participants who reported ≥2 exacerbations in the previous twelve months were considered as frequent exacerbators [28,36], and exacerbation episodes severe enough to require hospitalization for more than one day were considered hospital admissions.

### 4.4. Sputum and Blood Collection and Processing

Spontaneous sputum samples were collected and processed within 60 min on the day of the visit. Sputum quality was assessed according to Murray–Washington criteria [37] and only samples with >25 leucocytes per field were considered for the study. Sputum samples were processed for microbiome analysis as described previously [38].

Blood samples were obtained by peripheral venepuncture and placed in K_3_-ethylenediaminetetraacetic acid tubes for plasma analyses. All tubes were centrifuged at 1500× *g* for 15 min at 4 °C and supernatants were transferred to new tubes and stored at −80 °C until proteomic analyses, following previously specified protocols [39]. The approach covers 58 distinct protein biomarkers (cytokines, growth factors and acute phase proteins), which were assessed simultaneously [39]. The current study focused on peripheral blood IL-8 levels.

Bacterial 16SrRNA datasets from this study are accessible in the European Nucleotide Archive under the study PRJEB26773 with the sample numbers ERS2486515-609 (https://www.ebi.ac.uk/ena/browser/view/PRJEB26773 (accessed on 17 December 2025)).

### 4.5. Statistical Analyses

Categorical variables are expressed as absolute and relative frequencies and continuous variables as means and SD when the distribution is normal or as medians and IQRs otherwise. Correlations were assessed by Spearman rho correlation coefficients and comparisons between subgroups were assessed using Mann–Whitney U test for continuous variables. Patients reporting two or more exacerbations were considered frequent exacerbators and participants with one or more previous hospitalizations were considered as admitted patients. Spontaneous sputum samples were considered representative of bronchial secretions, and microbiome analyses were restricted to taxa that were present in ≥80% of the participants and showed RAs ≥ 0.1% [40,41], which were considered common taxa for the purposes of the study. Permutational analysis of variance [PERMANOVA] on unweighted UniFrac was used to compare beta-diversities of the microbial communities, and linear discriminant effect size (LEfSe) with a threshold value of 2.0 was used to explore differentially abundant taxa that explained discrepancies between groups [42,43]. IL-8 quantitation values were log-transformed to decrease their distribution skewness and make them suitable to parametric analysis [38].

Statistical tests used in the study were two-sided, and a *p*-value ≤ 0.05 was reported as statistically significant. Analyses were performed using the SPSS statistical software package version 29 (SPSS Inc., Chicago, IL, USA).

## 5. Conclusions

The present study shows that a clinical pattern of frequent exacerbations in COPD is related to a change in the bronchial microbiome, wider when hospital admissions are needed. The presence of *Haemophilus* was significantly associated with higher RAs of *Prevotella*, *Porphyromonas*, *Leptotrichia*, *Moryella*, *Megasphaera*, *Selenomonas* and *Bulleidia*, all part of the common bronchial flora. Higher RAs of *Porphyromonas*, *Leptotrichia* and *Selenomonas* were associated with lower IL-8 levels in peripheral blood. This relationship was also found for *H. parainfluenzae* as the main *Haemophilus* species identified in the studied COPD population. These findings suggest that part of the common components of the bronchial microbiome may have an anti-inflammatory role at a systemic level, a potential protective effect that would need additional prospective studies to be confirmed.

## Figures and Tables

**Table 1:** Demographic and clinical characteristics.

n	72
Age, mean (SD)	68 (7.9)
Gender (male), n (%)	64 (88.9)
Cumulative smoking (pack-year), median (IQR)	60 (45–80)
Postbronchodilator FEV_1_%, median (IQR)	44 (33–60)
Airflow limitation severity (GOLD criteria) [24], n (%)	
Mild	6 (8.3)
Moderate	20 (27.8)
Severe	35 (48.6)
Very severe	11 (15.3)
Clinical pattern (previous year)	
Exacerbations, median [IQR]	1 (0–2)
Frequent exacerbators (≥2/year), n (%)	20 (27.8)
Hospital admissions, median [IQR]	0 (0–1)
Hospital admission (≥1/year), n (%)	31 (43)

**Table 2:** Relative abundance of the genera detected. Only genera appearing with median relative abundances > 0.1% in more than 80% of the participants are shown.

Genera	Relative Abundance, Median [IQR]
*Rothia*	18.20 [9.62–30.08]
*Prevotella*	7.44 [3.27–14.97]
*Granulicatella*	4.42 [2.21–6.57]
*Fusobacterium*	2.23 [0.27–3.85]
*Porphyromonas*	1.97 [0.14–8.13]
*Streptococcus*	1.92 [1.25–3.22]
*Actinomyces*	1.80 [0.56–4.19]
*Pseudomonas*	1.35 [0.43–6.21]
*Veillonella*	1.00 [0.50–1.40]
*Atopobium*	0.69 [0.30–1.51]
*Oribacterium*	0.62 [0.19–1.23]
*Leptotrichia*	0.51 [0.10–1.71]
*Moryella*	0.35 [0.06–0.88]
*Campylobacter*	0.35 [0.10–0.76]
*Megasphaera*	0.25 [0.03–1.15]
*Bulleidia*	0.20 [0.05–0.85]
*Haemophilus*	0.19 [0.05–1.20]
*Selenomonas*	0.18 [0.04–0.61]
*Lactobacillus*	0.12 [0.01–1.03]
*Parvimonas*	0.11 [0.01–0.51]

**Table 3:** Relative abundances of taxa significantly associated with previous hospital admission in bronchial secretions (median [IQR]). Only taxa with median relative abundances > 0.1 in more than 80% of the participants are considered.

	Previous Admission		
	Absent	Present	Mann–Whitney U Test
*Prevotella*	9.32 [4.54–17.23]	4.66 [0.93–12.49]	0.031
*Moryella*	0.59 [0.12–1.23]	0.27 [0–0.68]	0.028
*Atopobium*	1.14 [0.42–2.294]	0.52 [0.24–1.13]	0.007
*Megasphaera*	0.36 [0.084–1.5]	0.1 [0–0.51]	0.016
*Parvimonas*	0.19 [0.02–0.6]	0.04 [0–0.16]	0.018
*Veillonella*	1.19 [0.61–1.68]	0.77 [0.41–1.17]	0.047
*Bulleidia*	0.45 [0.1–1.15]	0.08 [0.01–0.34]	0.003
*Selenomonas*	0.26 [0.07–0.64]	0.09 [0.01–0.36]	0.043

**Table 4:** Prevalence of identified *Haemophilus* species in the studied population (n = 72). Relative abundance median and interquartile range given for patients with the species present in the sample.

*Haemophilus* Taxa	Absolute Frequency	Relative Abundance Median [IQR]
*H. influenzae*	43	0.74 [0.36–2.52]
*H. parainfluenzae*	52	0.27 [0.17–0.48]
Other *Haemophilus* taxa	48	0.32 [0.2–0.54]
*Haemophilus* species unidentified	49	0.32 [0.22–0.59]

## Data Availability

Bacterial 16SrRNA datasets from this study are accessible in the European Nucleotide Archive under the study PRJEB26773 with the sample numbers ERS2486515-609 (https://www.ebi.ac.uk/ena/browser/view/PRJEB26773 (accessed on 17 December 2025)).

## Supplementary Materials

The following supporting information can be downloaded at: https://www.mdpi.com/article/10.3390/ijms27083416/s1.
