# Supplementary material for: Bronchial and Systemic Relationships of Haemophilus in Chronic Obstructive Pulmonary Disease
Source: Int J Mol Sci. 2026 Apr 10;27(8):3416. doi: 10.3390/ijms27083416 (PMC13115855; doi:10.3390/ijms27083416)
Supplement: Supplementary file 1 [file ijms-27-03416-s001.zip › ijms-4101227-supplementary.pdf]

BACK MATTER

BRONCHIAL AND SYSTEMIC RELATIONSHIPS OF HAEMOPHILUS IN CHRONIC OBSTRUCTIVE PULMONARY DISEASE

Supplementary material

Table S1 supplementary. - Relative abundance of the phyla detected. Only phyla appearing with median relative abundances >0.1% in more than 80% of the participants are shown.

Phylum	Relative abundance, median [IQR]
Firmicutes	29.1 [19.73-36.63]
Actinobacteria	24.67 [16.2-37.6]
Bacteroidetes	14.89 [5.25-25.48]
Proteobacteria	7.39 [3.3-18.49]
Fusobacteria	3.46 [0.56-6.02]
TM7	0.78 [0.06-1.87]

Table S2 supplementary. - Blood protein biomarkers. Results expressed as medians [IQR].

Protein abbreviations (pg/mL, except when specified)	Protein name	Median (IQR)
CCL21 / 6Ckine	C-C motif chemokine 21	17,049.19 [14,822.65-19,074.20]
CXCL13 / BCA-1	B cell-attracting chemokine 1	19.91 [13.96-29.70]
CCL27 / CTACK	Cutaneous T cell-attracting chemokine	1,677.36 [1,223.68-2,212.95]
CXCL5 / ENA-78	Epithelial-derived neutrophil-activating protein 78	451.31 [356.96-570.08]
CCL11 / Eotaxin	Eosinophil chemotactic protein	46.53 [37.17-59.74]
CCL24 / Eotaxin-2	Eosinophil chemotactic protein 2	244.09 [143.64-383.87]
CCL26 / Eotaxin-3	Macrophage inflammatory protein 4-alpha	37.65 [32.43-45.67]
CXCL6 / GCP-2	Granulocyte chemotactic protein 2	20.27 [13.55-30.98]
GM-CSF	Granulocyte colony macrophage stimulating factor / Colony-stimulating factor 2	15.23 [13.48-17.6]
CXCL1 / Gro-alpha	Growth-regulated protein Alpha	197.9 [171.01-278.82]
CXCL2 / Gro-beta	Growth-regulated protein beta	220.78 [142.92-322.29]
CCL1 / I-309	T lymphocyte-secreted protein I-309	58.52 [49.56-67.06]
CXCL11 / I-TAC	Interferon-inducible T-cell alpha chemoattractant	9.03 [6.21-12.99]
IFN-gamma	Interferon gamma	6.29 [5.46-7.35]
IL-1	Interleukin 1	0.82 [0.49-1.41]
IL-2	Interleukin 2	10.61 [7.28-13.85]
IL-4	Interleukin 4	4.42 [3.41-5.37]
IL-7	Interleukin 7	10.94 [5.22-18.33]
CXCL8 / IL-8	CXCL motif chemokine-ligand 8	10.65 [9.31-13.04]
IL-9	Interleukin 9	1,118.93 [1,011.75-1,223.09]
IL-10	Interleukin 10	6.79 [5.65-8.31]
IL-16	Interleukin 16	85.02 [68.71-112.51]
CXCL10 / IP-10	C-X-C motif chemokine ligand 8 / Interferon gamma-induced protein 10	293.23 [196.74-383.39]
CCL2 / MCP-1	C-C motif chemokine ligand 2	13.96 [11.46-20.4]
CCL8 / MCP-2	Monocyte chemotactic protein 2	24.95 [19.93-31.9]
CCL7 /MCP-3	Monocyte chemotactic protein 3	52 [35.79-87.16]
CCL13 / MCP-4	Monocyte chemotactic protein 4	28.28 [21.57-43.69]
CCL22 / MDC	Macrophage-derived chemokine	420.06 [260.11-531.48]
MIF	Macrophage migration inhibitory factor	25,789.74 [20,696.67-32,103.74]
CXCL9 / MIG	Monokine induced by interferon gamma	52.46 [35.89-76.03]
CCL3 / MIP-1alfa	Macrophage inflammatory protein 1 alpha / C-C motif chemokine ligand 3	1.2 [0.9-1.89]
CCL15 / MIP-1delta	Macrophage inflammatory protein 1 delta	2,311.91 [2,065.12-2,693.63]
CCL19 / MIP-3beta	Macorphage inflammatory protein 3 beta	146.56 [120.89-185.59]
CCL23 / MPIF-1	Myeloid progenitor inhibitory factor 1	308.9 [205.59-433.40]
CXCL16 / SCYB16	Small-inducible cytokine B-16	502.65 [404.73-559.33]
CXCL12 / SDF1alphabeta	Stromal cell-derived factor 1	1,747.03 [1,576.43-1,993.63]
CCL25 / TECK	Thymus-expressed chemokine	552.14 [426.77-759.73]
TNF-alpha	Tumor necrosis factor alpha	78.64 [75.6-85.40]
FGF2	Fibroblast growth factor 2 / Basic fibroblast growth factor	24.4 [21.67-29.3]
G-CSF	Granulocyte colony stimulating factor / Colony stimulating factor 3	72.5 [53.79-97.63]
PDGFB	Platelet-derived growth factor subunit B	205.45 [125.77-364.53]
CCL4 / MIP-1beta	C-C motif chemokine ligand 4 / Macrophage inflammatory protein 1-beta	291,71 [259,28-319,96]

CCL5 / RANTES	C-C motif chemokine ligand 5	7,442.81 [3,949.80-9,483.7]
A-2-M (ng/mL)	Alpha-2-macroglobulin	2,038,700 [1,787,150-2,568,200]
Haptoglobin (ng/mL)	Haptoglobin	599,383.22 [438,869.46-966,551.13]
C-reactive protein (ng/mL)	C-reactive protein	3,835.2 [2,170.91-7,517.46]
SAP (ng/mL)	Serum amyloid P-component	50,396.61 [41,604.23-60,378.07]

Table S3 supplementary. - Relationships between common respiratory taxa and protein biomarkers. Spearman rho coefficient between relative abundance of each taxon and peripheral blood level of the biomarker. In bold, statistically significant relationships (*p<0.05 / **p<0.01). Only biomarkers with one or more statistically significant relationships are displayed.

Protein	Taxa								
	H.influenzae	*H.parainfluenzae*	*Prevotella*	*Porphyromonas*	*Leptotrichia*	*Moryella*	*Megasphaera*	*Bulleidia*	*Selenomonas*
6Ckine	-0.1411	**0.2522***	-0.0249	-0.0205	0.0229	0.0473	0.0384	0.0826	-0.0660
IL-8	0.0930	**-0.3230****	-0.1267	**-0.3145****	**-0.3255****	-0.1162	-0.2184	-0.2082	**-0.2658***
I-TAC	0.0814	-0.0167	**0.2406***	0.0881	0.1234	0.1340	0.1005	0.1284	0.1961
SCYB16	-0.1123	-0.0906	**-0.2942***	**-0.2380***	-0.0771	-0.0149	-0.1726	-0.1291	-0.0652
SDF1alfabeta	-0.0390	0.1098	**-0.2433***	-0.1497	-0.0002	-0.0014	-0.0611	-0.0512	-0.0310
TECK	0.02198	0.0338	**-0.2610***	-0.1935	-0.0944	0.0105	-0.1314	.-0.1377	-0.0808
PDGFB	-0.1371	0.0281	**0.2524***	0.0467	0.0736	**0.3316****	0.2031	**0.3233****	0.1413
Haptogobin	-0.0967	-0.1189	0.2219	0.1494	**0.3836****	0.0492	-0.0748	0.1185	0.2180
SAP	0.1913	0.1913	0.2134	0.2014	0.1621	0.1586	**0.2525***	0.1606	0.0234
